# Determination of Selected Texture Features on a Single-Layer Grinding Wheel Active Surface for Tracking Their Changes as a Result of Wear

**DOI:** 10.3390/ma14010006

**Published:** 2020-12-22

**Authors:** Anna Bazan, Andrzej Kawalec, Tomasz Rydzak, Paweł Kubik, Adam Olko

**Affiliations:** 1 Faculty of Mechanical Engineering and Areonautics, Rzeszow University of Technology, Powstancow Warszawy 12, 35-959 Rzeszow, Poland; ak@prz.edu.pl (A.K.); t.rydzak@prz.edu.pl (T.R.); p.kubik@prz.edu.pl (P.K.); 2 Pratt&Whitney Rzeszow, Hetmańska 120, 35-001 Rzeszow, Poland; adam.olko@prattwhitney.com

**Keywords:** electroplated grinding wheel, grinding wheel wear, grinding wheel surface texture

## Abstract

Measurements of the active surface microgeometry of the grinding wheel by contact and optical methods are commonly used to obtain a cloud of points representing the surface of the examined tool. Parameters that can be determined on the basis of the above-mentioned measurements can be universal parameters, which are commonly used to assess the geometric structure of a surface or parameters taking into account specific properties of the grinding wheel active surface (GWAS) structure. This article proposes a methodology for determining the average level of binder, which allows the definition the cut-off level required to separate from the measurement data: (i) the areas representing grains, (ii) the areas of gumming up of the grinding wheel, and (iii) deep cavities in approximately the same places on the investigated grinding wheel, regardless of the degree of its wear. This, in turn, allows one to track changes in characteristic parameters computed from measurements of texture in the above-mentioned areas due to different GWAS wear processes. The research was based on the analysis of data obtained from measurements of single-layer grinding wheels using the replica technique. The adopted measurement methodology enables measurement of approximately the same (94% coverage) areas of the GWAS at four stages of grinding wheel operation. Errors that were computed related to the determination of the volume of abrasive on the GWAS at various stages of wear using the developed methodology were lower, on average, by 48% compared to the automatic recognition of islands made with a commercial software.

## 1. Introduction

A grinding wheel’s surface microgeometry belongs to the most important features influencing interactions between any grinding wheel and a work-piece during grinding. It decides, among other things, the magnitude of such parameters associated with the grinding process as grinding force, energy, and grinding temperature [1,2,3]. Thereby, a grinding wheel’s geometry influences the grinding process flow and its effects, as well as the quality of the manufactured surface.

Microgeometry, apart from the types and properties of abrasive grain materials and binders and the features of the grinding wheel structure, is one of the important factors determining the cutting ability of this tool, i.e., the ability of the grinding wheel to remove machining allowance [4,5]. Microgeometry, in turn, depends on the topography of the grinding wheel’s active surface (GWAS) and geometric features of the abrasive grains, e.g., their height, slope steepness, or characteristic angles [6,7,8,9]. To describe each of these features quantitatively, several parameters can be used, which are determined in various ways and characterized by a better or worse ability to characterize the considered feature. For example, the height of the grains on the GWAS can be represented by the parameter *Sq* (root mean square height) [7,8], the mean height of the elevations above the cut-off point determined to the highest elevation [10], mean or maximum height of elevations above the designated mean area [11], and medium or maximum height of the motifs determined using motif analysis [12]. Information about the GWAS microgeometry is important during the entire time of grinding wheel operation.

The cutting potential of single-layer grinding wheels (SLGWs) is associated with a very limited amount of abrasive. Their cutting properties cannot be restored by dressing. However, they are often used for grinding products that are subject to high dimensional and shape requirements and made of difficult-to-machine materials, e.g., integrally bladed rotors made of nickel superalloy [13,14] or high-hardness steel gears [15,16,17]. Therefore, any instability in a manufacturing process implementing such grinding wheels can result in significant financial losses. This is one of the main reasons for insightful testing of SLGWs, including their topography.

Quantitative information on the active surface of the grinding wheel is most often obtained by analyzing 2D images, obtained with, e.g., a scanning electron microscope (SEM) [7,18,19,20,21,22,23,24], atomic force microscope (AFM) [25,26], or optical microscope [18,27,28,29,30,31,32,33,34], and from 3D measurements of surface topography using, e.g., contact profilometers [7,8,35,36], confocal microscopes [12,37,38], and interferometers [37,39,40,41]. Indirect measurement methods, such as measuring the weight of the grinding wheel before and after grinding, allow one to specify the volume of the grinding tool consumed during the process [24,42,43] and to determine the grinding ratio *G*. Unlike the indirect methods, direct observation and measurement methods of the GWAS topography allow one to obtain data with a much wider range of applications. However, the interpretation of collected and computed data is still difficult and relevant. Visual assessment of the microscopic GWAS images enables one to distinguish between static and active grains and to detect various forms of grinding wheel wear. Thanks to this, some researchers determine some quantitative parameters directly on the basis of the above-mentioned 2D images. These parameters are, e.g., the number of active grains per unit area and the number of grains torn out of the binder per unit area [6,18,32,44,45].

The developed methods of image analysis applied to 2D GWAS views allow one to determine, among other things, the distance between the grains, the surface area and the percentage of the surface area of the grains and areas of gumming up of grinding wheels [46], and the maximum and minimum diameter of the Feret grains [47]. Compared to a microscopic image analysis performed directly by a researcher, computer image analysis methods allow larger areas of the GWAS to be analyzed in less time. On the other hand, direct analysis by a researcher enables precise determination of the number of grains and their boundaries. In addition, the places where some grains have been torn out from SLGWs and cavities in the bond have formed can be relatively easily recognized by a researcher. It becomes very difficult or even impossible to determine them at all using known image analysis methods [48].

The determination of the height and volume parameters of texture is possible from the results of 3D measurement of GWAS topography. Several surface texture (ST) parameters can be calculated according to the ISO standards [49,50] or the European Union report [51]. They require, however, insightful analysis. Nguyen and Butler et al. [7,8] interpreted the parameter *Sds* (summit density) as the density of cutting edges. The parameter *Ssc* (mean summit curvature) was, however, associated by them with the radius of the rounding of the grain, and thus with the sharpness of the grains. The same interpretation of the parameters *Sds* and *Ssc* was used by Yan et al. [40]. Moreover, the parameter *Sdq* (root mean square gradient) was supposed to indicate the angles of the slopes of the grains. The authors of Ref. [40] also associated the sum of the parameters *Vvc* (core void volume) and *Vvv* (dale void volume) with the volume of space on the GWAS where chips can collect. Wang et al. [52] observed the aforementioned parameters *Ssc* and *Sdq* as well as the peak–peak height (*Sz*) while examining the wear of grains on the abrasive belt. Kapłonek and Nadolny [53,54] indicated the suitability of grinding wheels for the assessment of the surface of grinding wheels in terms of their sticking and abrasion of such ST parameters as *Sdr* (surface development factor) and *Sk* (surface core height). Vidal et al. [38] investigated several selected surface texture parameters, of which *Svk* showed the greatest changes due to the GWAS wear, and in the case of dressing, the parameter *Sa* changed the most.

The above-mentioned ST parameters are related to different GWAS features only to a limited extent. The analysis of elevations above the cut-off level (particles) and pits or cavities below the cut-off level (pores) can provide important information about the condition of the GWAS. With the appropriate cut-off level, the particles can represent areas associated with abrasive grains or areas of sticking, while pores can represent voids or pores in the binder. Therefore, the determined parameters of particles and pores have a direct reference to grinding wheel design.

The distribution and shape of abrasive grain areas above a certain level may indicate the activity of abrasive grains. They may be used to evaluate the wear processes of the GWAS and to select appropriate machining parameters. For example, in order to evaluate the cutting potential of a GWAS, Kacalak et al. developed, on the basis of the Teager–Kaisear energy operator [55], a parameter depending on the height and sharpness of the slopes of the tops of grains above the level dependent on the area of the average measured topography [56]. On the other hand, the parameters of the pores are factors that influence, among other things, the lubrication and cooling conditions during grinding. Hence, they can be used to model the phenomena related to the flow of cutting fluid through the grinding zone [57,58].

The natural separation boundary for particles and pores in the case of SLGWs is the binder, and that approach was used by Setti et al. [59]. However, it is difficult to automatically determine the level of separation between the grain and the binder on measured GWAS topographies [37,60]. Ismail et al. presented a method for determining a reference surface that would represent the level of binder using the so-called inverse method (reversal method), which consists of measuring the same surface in several rotational positions [12]. The authors assessed that this method proved to be effective for 66% of the investigated areas. In the remaining cases, it was not possible to determine the reference area due to the low repeatability of the ST measurements performed.

**Figure 1:**
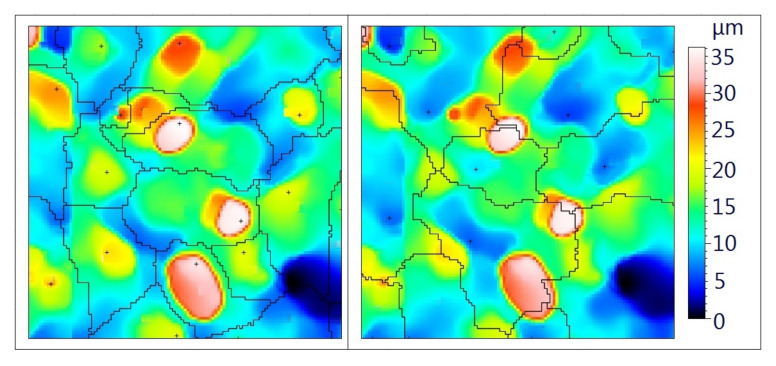
Segmentation of motifs associated with hills (**left**) and valleys (**right**).

In their investigations, Kacalak et al. applied [9,10] watershed segmentation and the related analysis of motifs to determine the parameters of abrasive grains. The separation of motifs can be done in two ways—to recognize motifs related to either valleys or hills. In the first case, a single motif consists of the valleys and the surrounding area. In the second case, the motif covers the hill with its immediate surroundings (Figure 1). This means that in both cases, the motifs do not only contain data on grinding wheel grains or deep cavities, but also some more information on surface texture. The authors of Ref. [61] also used the analysis of motifs in the study of grinding wheel topography.

Various segmentation methods from three-dimensional data for different types of surfaces were analyzed in [62,63,64,65]. The problem of determining the critical points necessary to conduct spatial segmentation is discussed in [66]. It should be remembered that the issue of segmentation does not only refer to surface macrogeometry. It is a technique with a wider application and is also used to analyze macrogeometry [67].

In the Image Metrology’s commercial Scanning Probe Image Processor (SPIP^TM^) software for ST analysis, watershed segmentation can isolate entire motifs or only elevations and pits without their surroundings. The main disadvantage of watershed segmentation for ST assessment of GWASs is the difference of the cut-off levels of individual extracted elements in both the analysis of motifs and the recognition of hills and cavities. Consequently, the height and volume parameters are not determined from a common reference surface. Therefore, it is not possible to compare the volume or the maximum height of two selected abrasive grains.

Commercial programs for the analysis of surface texture, such as SPIP by Image Metrology or MountainsMap by DigitalSurf, allow one to analyze particles and pores segmented with a single surface. However, it is important that particles and pores on a GWAS are determined with the cut-off level independent from the degree of GWAS wear. In order to always cut off and analyze the same parts of the considered ST features, the cut-off level should be selected in a way that keeps the same or approximately the same place for a given grain independently from sticking or recess processes.

The cut-off level can be determined within SPIP or DigitalSurf at a user-defined distance from the reference element, which may be, e.g., the average area or the highest or lowest measured point. Self-determination of the reference level by the user allows full control over it. The cut-off level can be guided by visual inspection of the measured topography. Moreover, the result is not dependent on algorithms that are unknown to the user in the computational (mathematical) layer. On the other hand, the independent determination of the reference level by the user is more labor-intensive and time-consuming in analysis of a large number of measured surfaces.

To reduce such effects, the extraction of the considered features from the measured topographies should be automated. Of the two above-mentioned software packages, only SPIP has an automatic cut-off function. The conducted research described in Section 3 showed, however, that the use of this function did not lead to satisfactory results related to the separation of areas corresponding to the abrasive grains. For this reason, a proprietary algorithm for calculating cut-off levels for particles and pores was developed in the paper. It was implemented in the SPIP software as a plug-in. It enables determination of the cut-off level automatically and processing of many measured examples of GWASs, as well as the determination of the required ST parameters of investigated GWASs in batch processing.

In Section 7, the results of tests related to the use of the developed algorithm for determining the cut-off level and separation of areas associated with abrasive grains, areas of sticking, and cavities in the binder from the measured texture of GWASs are shown. The analyses were done for one single-layer electroplated cubic boron nitride (cBN) grinding wheel at various stages of grinding wheel wear. The paper ends with conclusions drawn from the research and bibliography analysis.

## 2. Research Methodology

The surface topography studies concerned the active surfaces of 17 SLGWs with a nickel bond applied by electroplating with a cBN abrasive with grain number B35 (average grain diameter of $d_{g}=35\;\mu m$). The grinding wheels had a conical shape with a maximum diameter of $d_{s}=100$ mm and a cone angle of $140^{\circ }$.

The ground items were made of high-alloy Pyrowear 53 steel after thermo-chemical treatment, with a surface layer hardness of 81 HRA. The surface grinding processes were carried out on a Fortis grinder by Michael Deckel in the presence of grinding oil. Each grinding wheel was operated with a different set of adjustable parameters until it was completely worn (manifested by an intensive increase in grinding force) or until the specific volume of material was removed ($V^{\prime }=2652\;{\mathrm{mm}}^{3}/\mathrm{mm}$). The adjustable parameters of the grinding process were changed in the following ranges:

- Grinding speed (for diameter $d_{s}=100\;\mathrm{mm}$): $v_{s}=20$–40m/s, which corresponds to the rotational speed range of the grinder spindle: n=4000–8000rev/min;
- Feed speed: $v_{w}=1000$–7500mm/min;
- Grinding depth: $a_{e}=7$–30μm (0.2–$0.86\cdot d_{g})$.

**Figure 2:**
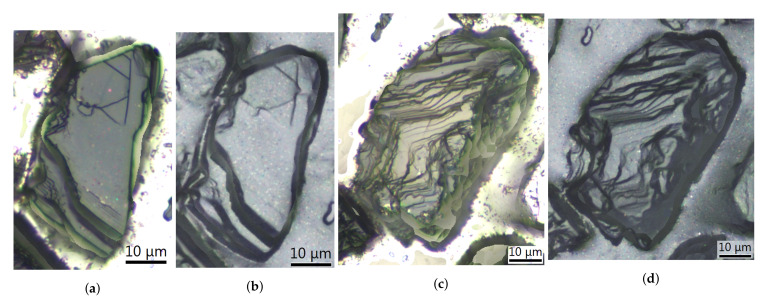
Views of the grains observed directly on the InfiniteFocus microscope (**a**,**c**) and the same grains mapped by the replica (**b**,**d**).

The active surface topography of each of the tested grinding wheels was measured several times at different stages of grinding wheel wear. The GWAS tests were planned after the removal of the following specific volume of material $V^{\prime }$ [${\mathrm{mm}}^{3}/\mathrm{mm}$]: 0 (new grinding wheel), 204 or 272, 408 or 476, 1224 or 1360, 2652, or when the grinding wheel was worn out. Replicas were used to map the GWAS topography. The use of replicas was due to two reasons. First, by using replicas, it was not necessary to remove the grinding wheel from the machine and tool holder to measure GWAS topography. This significantly shortened the testing time and also improved the stability of the grinding wheel’s operating conditions throughout its lifetime. The second reason was related to difficulties with direct measurement of the GWAS on the InfiniteFocus microscope. The areas of bonding near the grains were of complex shape and intractable to measure. The problems of performing direct measurements on the grinding wheel were probably caused by big differences in color between grains and the bond. It was not possible to set such measurement conditions, i.e., brightness and contrast, to lighten up grains and the bond properly at the same time. The replicas were made using the Struers RepliSet system. According to information provided by the manufacturer, the material of the replicas was a black silicone rubber with the ability to reproduce details above 0.1μm. In order to visualize the precision of the replication method, the views of some grains obtained directly from the grinding wheel with the use of the InfiniteFocus microscope were compared to the related views from the replicas. It can be seen in Figure 2 that only the smallest details located on the investigated grains, such as some lines from cleavage planes, were not mapped.

Thanks to the use of replicas, in order to test the microgeometry of the grinding wheel, it was not necessary to remove the tool (grinding wheel) from the machine tool. This reduced the work and time consumption of the research. It is worth mentioning that replicas can also be helpful in the case of measurements of large grinding wheels that may not fit on the measuring device; e.g., a profilometer or microscope.

**Figure 3:**
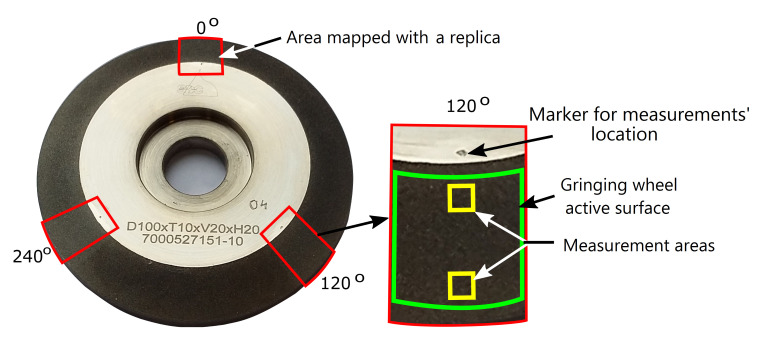
Replicas’ production sites and measuring surfaces on the grinding wheel active surface (GWAS).

An Alicona InfiniteFocus microscope with a ×20 lens was used to measure the topography of the GWAS replicas. At each of the tested grinding stages, six areas that were approximately the same with dimensions of 2.35mm × 2.59mm were measured. The measured surfaces were spaced at $120^{\circ }$ around the axis of rotation of the grinding wheel (Figure 3). Two surfaces with different radial positions were measured at each angular position. The measurement parameters of the GWAS replicas are presented in Table 1.

### Positioning of Measuring Surfaces

As mentioned in the introduction, the GWAS topography analysis can be very useful in tests related to wear, and then in the supervision of the cutting properties of grinding wheels. In order to obtain the necessary data, the GWAS measurement should be carried out at various stages of the grinding wheel wear, i.e., after removing a different volume of material. Wear information of the best quality was obtained by continuously measuring the same places on the grinding wheel. Changes in the calculated GWAS parameters at various stages of wear resulted not only from the wear itself, but also from the variance of the values of these parameters within the GWAS. The analysis of the grinding wheel wear based on the measurement of various areas of the GWAS after the removal of subsequent volumes of material only gave the possibility of tracing the generalized changes in the topography parameters within the GWAS. When constantly comparing the same GWAS areas, the changes observed in the GWAS parameters resulted primarily from the wear of the grinding wheel. The conducted analyses were not burdened with an error resulting from possible changes of these parameters within the GWAS. In addition, by measuring the same places on the grinding wheel, it was possible to determine how specific abrasive grains were worn out.

In order to measure the GWAS topography in approximately the same places at different stages of wear, marks were made on the face of the grinding wheel with a vibrating pen in the form of small pits. These characteristic points were spaced around the axis of rotation of the grinding wheel every $120^{\circ }$ (Figure 3). They indicated the places where replicas were to be made. At the stage of measuring the GWAS topography mapped with replicas on the InfiniteFocus microscope, the markers were used for the initial orientation of the measurement surfaces. The precise orientation of the measurement surfaces was based on a selected characteristic detail of the GWAS, such as an abrasive grain with a distinctive shape.

**Figure 4:**
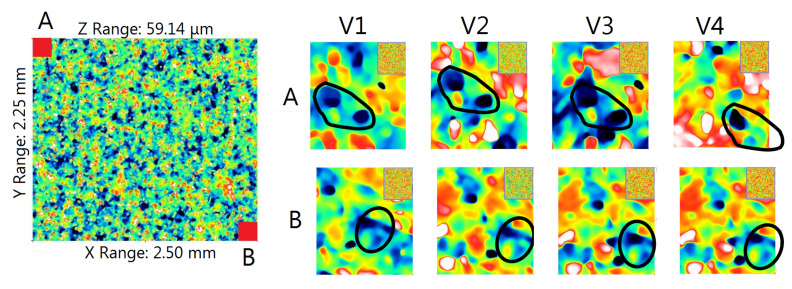
Views of the opposite corners of the topography maps, marked as A and B (left), obtained as a result of measuring corresponding areas on four replicas of V1÷V4 made at different stages of grinding wheel wear (right).

The reproducibility of the positioning of the measurement surfaces—to ensure the measurement of the GWAS topography in approximately the same places at different stages of the study—was checked for one randomly selected measurement spot on the grinding wheel, which was considered the most worn after visual inspection of the topography maps. The research on the appropriate positioning of the measuring surfaces is illustrated in Figure 4. Four topography maps (marked as V1, V2, V3, and V4) obtained as a result of measurements carried out on four replicas made at different stages of grinding wheel wear were analyzed. From each of the topography maps with dimensions of 2.25mm × 2.50mm, two areas (A and B) of approximately 0.22mm × 0.24mm were located in opposite corners of maps. Then, four views of the A areas (coming from four replicas) and four views of the B areas were compared with each other. To make the comparison easier, in views A and B, the characteristic fragments of the topography that were found on the corresponding views were selected. In Figure 4, these fragments are outlined. The comparison of the opposite corners of the four topography maps allowed the identification of the area common to all analyzed maps.

In the four analyzed topography maps showing fragments of the GWAS with area 5.625 mm^2^, the surface area of the repeating area (the area common to all four maps) was approximately 5.29 mm^2^. It accounted for approximately 94% of the measuring area.

## 3. Automatic Determination of the Cut-Off Level in the SPIP 6.4.2 Software

In order to obtain data on particles and pores separated from the measured GWAS topography, which would be most useful during the analysis of the grinding wheel wear, two conditions were met: At different stages of wear, the same places on the grinding wheel were constantly measured, and the cut-off levels for particles and pores were determined in such a way that they cut off the same grain fragments, areas of gumming up of the GWAS, and cavities. In this part of the article, the results of the study showing the separation of particles using the automatically selected cut-off level in the SPIP 6.4.2 software will be presented.

**Figure 5:**
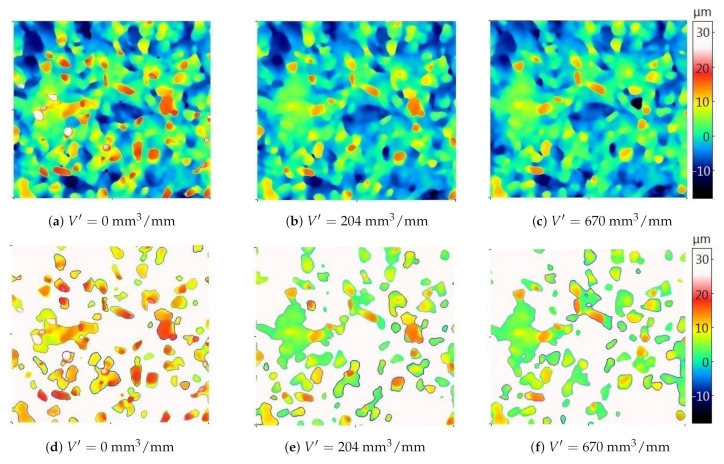
Maps of the new (**a**) and used (**b**,**c**) grinding wheel topography and the corresponding areas of particles above the automatically determined cut-off level (**d**–**f**). The color palette is the same for all six images. Grinding parameters: $v_{s}=30$ m/s (for maximum grinding wheel diameter $d_{s}=100$ mm), tangential feed speed $v_{w}=4250$ mm/min, grinding depth $a_{e}=20\;\mu m$.

Figure 5 shows a fragment of the measured active surface topography of the new grinding wheel (the actual material loss $V^{\prime }=0\;{\mathrm{mm}}^{3}/\mathrm{mm}$), that found after removing the specific material volumes equal to $V^{\prime }=204\;{\mathrm{mm}}^{3}/\mathrm{mm}$, and that found after complete wheel wear ($V^{\prime }=680\;{\mathrm{mm}}^{3}/\mathrm{mm}$). The total wear of the grinding wheel was associated with an intensive increase in the grinding force. On the measured surface of the new grinding wheel, the cut-off level was clearly “higher” than on the measured topographies of the used grinding wheel. In other words, in the case of a used wheel topography, more areas belonging to the bond were above the cut-off level than was the case with the wheel before its operation. The results for the automatically determined (AT) cut-off level are also presented in Section 6.

The percentage of the surface particles presented in Figure 5 was A%=22.19% for a new grinding wheel, A%=22.81% for the specific material loss $V^{\prime }=204\;{\mathrm{mm}}^{3}/\mathrm{mm}$, and A%=22.20% for a completely worn wheel. The analysis of the replica photos showed that there was no chip build-up on the GWAS. Therefore, increasing the proportion of particles (corresponding to abrasive grains) on the grinding wheel in the used condition compared to the new grinding wheel is illogical. The analysis of the percentage share of particles confirms the visual assessment of the areas above the cut-off level and the conclusion that the automatically selected cut-off level changes significantly depending on the degree of wear of the grinding wheel. A quantitative measure of the error in determining the cut-off level for abrasive grains at different stages of wear using the automatic threshold (AT) function is presented in Section 5.

## 4. Calculation of the Average Level of the Binder

**Figure 6:**
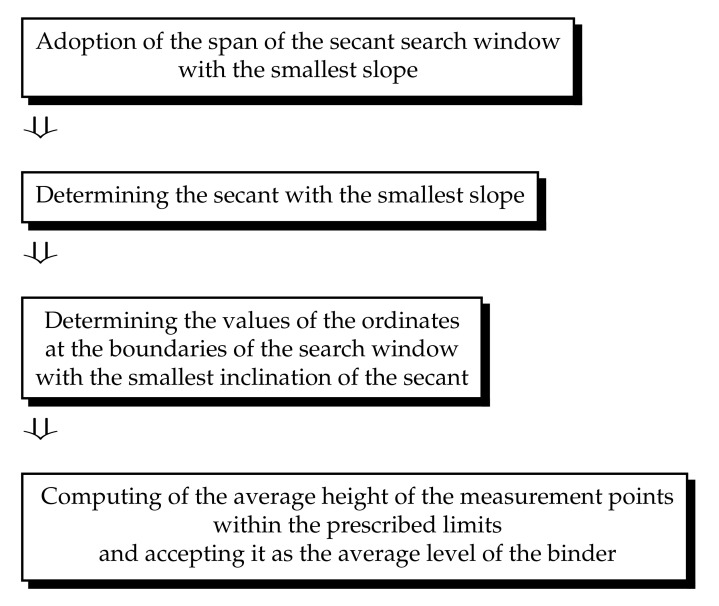
Algorithm for determining the average level of the binder.

**Figure 7:**
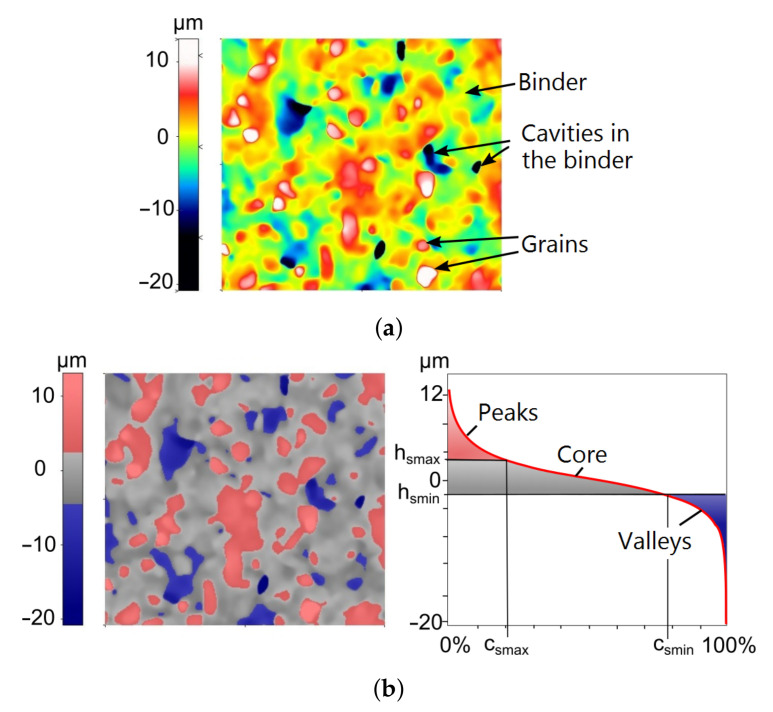
The areas of grains, binder, and cavities in the binder (**a**), as well as their corresponding areas on the bearing area curve (**b**) [68].

The function of automatic determination of the cut-off level for pores and particles available in the SPIP 6.4.2 program for the analyzed topographies of the tested grinding wheels did not meet the expectations. For this reason, a proprietary algorithm for determining the cut-off level was developed, which depended on the level of binder on the grinding wheels. The main idea of the algorithm is based on the analysis of the bearing area curve (BAC), also called the Abbott–Firestone curve, and was already presented in Ref. [68]. It is also briefly illustrated in Figure 6 and Figure 7.

The measurement points on the BAC related to the binder correspond particularly to the core area, i.e., the central part of the curve. Abrasive grains and deep pits after grain extraction on the BAC correspond, respectively, to the areas on the left side of the curve with the lowest values of the material share and the right side of the curve with the highest values of the material share (Figure 7). Compared to the grains and grain pits, the point height differences within the binder are small. The developed concept assumes that in order to determine the average level of the binder (average level, because the binder is related to a certain range of ordinate values, and not only a single value), the tangent line to the BAC with the smallest slope at a specific span of the search window should be determined. An analogous secant with a window span of 40% is used to calculate the topography parameters *Sk*, *Spk*, and *Svk* according to the [49] standard. The algorithm for determining the average level of the binder is shown in Figure 6.

Based on the analysis of the surface share of the binder on the maps of the topography of the tested grinding wheels, in Ref. [68], the span of the search window for the smallest inclination was assumed for 40% of the material share. In the described research, it was checked how the width of the search window affects the determined value of the average level of the binder. For this purpose, 10 measured GWAS topographies with different degrees of grinding wheel wear were randomly selected. On each of these topographies, the average level of the binder was determined with a window span of 20% to 70% of the material content in 10% steps. Group differences were tested using the Wilcoxon signed-rank test with a significance level of α=0.05.

It was noticed that the seven study groups could be divided into two sets. The first contained groups related to the window spans of 20%, 30%, and 40% (set 20–40), and the second contained groups related to the window spans of 50%, 60%, and 70% (set 50–70). Within each set, the groups showed no statistically significant differences. On the other hand, significant differences were observed when comparing groups from different collections.

On average, in the set 50–70, the determined binder level was 0.17μm higher than in the set 20–40. This was probably due to the fact that with the larger slope search windows, with the smallest inclination, those windows also contained points belonging to the grains. For this reason, a search window equal to 40% of material share was adopted for further research.

## 5. Comparison of Results Obtained Using Automatically Determined (AT) Cut-Off Level and the Developed Algorithm (OA)

In order to compare the effects of extracting grain areas using the automatically determined (AT) cut-off level and our own developed algorithm (OA), 10 pairs of measurement data were used. Each pair was related to the same measurement area, but at a different stage of wear. For each pair, the level of particle cut-off was manually determined, so that for a given pair, it ran in the same place and cut off analogous grain fragments.

**Figure 8:**
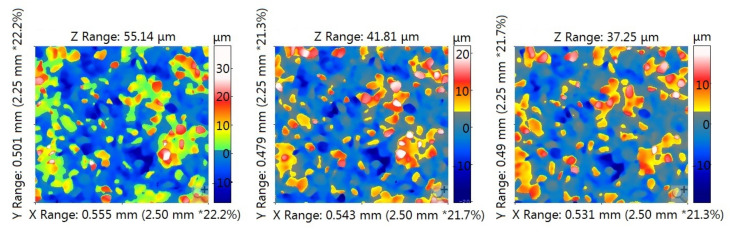
View of three maps analyzed at the same time to manually determine the cut-off level at different stages of wear; the area below the cut-off level is marked as blue.

Manual determination of the cut-off level included simultaneous analysis of 2–3 surface maps established at the same location on the grinding wheel and at different stages of its wear. One of the analyzed 2D maps was always a new grinding wheel surface map. On each map, a characteristic arrangement of details was selected—a pattern of several grains or a cavity with a characteristic shape. Views of the maps were enlarged to dimensions of about 0.5 mm × 0.5 mm because individual grains and cavities were clearly visible in such a window. The color palette on each map was always chosen to be similar to other analyzed maps (Figure 8). Thanks to that, it was easy to find and compare individual grains on different maps. After such preparations, the cut-off level from the cavities to the hills was manually “moved in space” and set at the level ensuring that most of the binder was below it. The process of moving the cut-off level can be compared to flooding the surface with water. For determining the cut-off level, the map of a new grinding wheel was used as the reference map. Given that all maps showed distinctive grains, attempts were made to determine the cut-off level in such a way that the grains were separated from their surroundings at the same level in all cases.

For all data, the volume of particles above the cut-off level determined manually ($V_{m}$), automatically ($V_{\mathrm{AT}}$), and with the use of the developed algorithm ($V_{\mathrm{OA}}$) was calculated. Then, for pairs of surfaces and a given method of determining the cut-off level, the difference between the volume of particles at the earlier and later stages of wear (ΔV) was calculated. The quality of the effects of the automated computation of the threshold AT and OA cut-off methods was determined by comparing $\Delta V_{\mathrm{AT}}$ and $\Delta V_{\mathrm{AT}}$ to $\Delta V_{m}$. The error (err) of the method was assumed to be the difference between the value obtained when determining the manual cut-off level ($\Delta V_{m}$) and the value (ΔV) related to this method. The absolute error $\left({\mathrm{err}}_{\%}\right)$ of the method was determined using the formula: ${\mathrm{err}}_{\%}=\mathrm{err}/\Delta V_{m}$.

For all 10 pairs of surfaces, the error in determining the cut-off level was lower with the use of the developed algorithm than with the automatic method. The quality of the cut-off level determination improved, on average, by 48% (Table 2). The maximum improvement was over 125%. It is also worth noting that the third error quartile in the OA method is smaller than the minimum error determined for AT. Therefore, it can be concluded that the developed algorithm allows the separation of the same grain areas at different stages of grinding wheel wear to a much better extent.

The average error in determining the cut-off level using the developed algorithm was over 9%. It is therefore appropriate to continue to search for and refine methods for determining the cut-off level to separate grain regions and pits on the measured GWAS topographies. It should be remembered that the manual method is also not ideal and is burdened with an error resulting from the subjective determination of the cut-off level, depending on the researcher.

**Figure 9:**
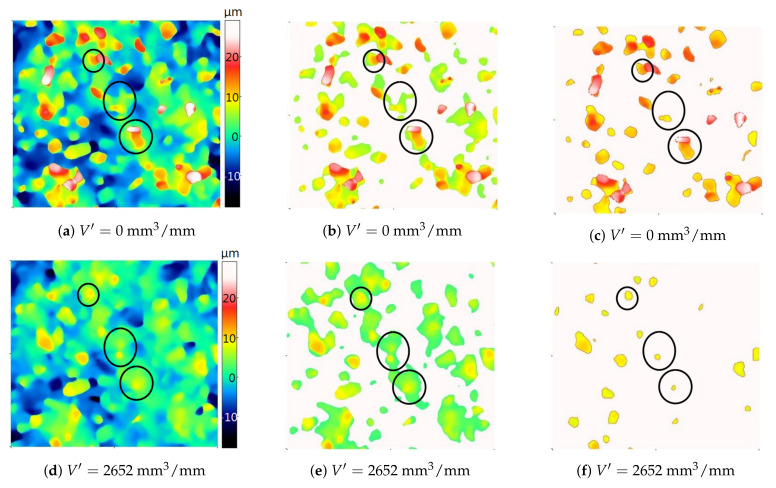
Maps of the new (**a**) and used (**d**) grinding wheel topography and the corresponding areas of particles above the automatically determined cut-off level (**b**,**e**) and the cut-off level determined by the developed algorithm (**c**,**f**). The color palette is the same for all six images. Grinding parameters: $v_{s}=40\;m/s$ (for the maximum grinding wheel diameter $d_{s}=100\;\mathrm{mm}$), tangential feed speed $v_{w}=7500\;\mathrm{mm}/\min$, grinding depth $a_{e}=20\;\mu m$.

**Figure 10:**
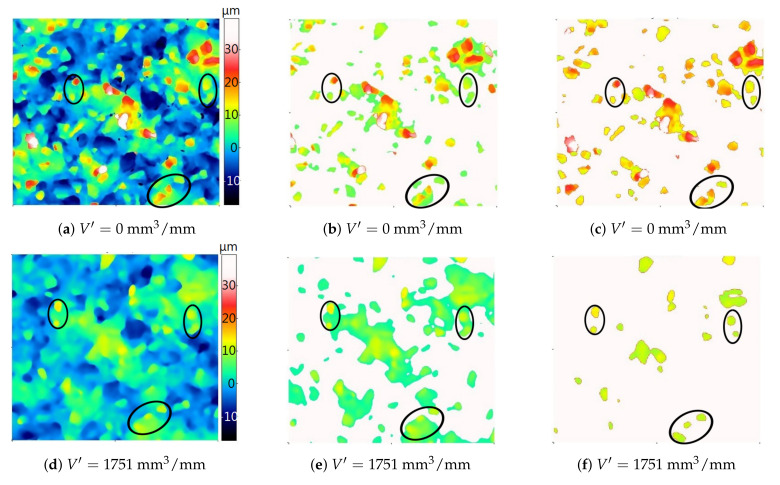
Maps of the new (**a**) and used (**d**) grinding wheel topography and the corresponding areas of particles above the automatically determined cut-off level (**b**,**e**) and the cut-off level determined by the developed algorithm (**c**,**f**). The color palette is the same for all six images. Grinding parameters: $v_{s}=20\;m/s$ (for the maximum grinding wheel diameter $d_{s}=100\;\mathrm{mm}$), tangential feed speed $v_{w}=4250\;\mathrm{mm}/\min$, grinding depth $a_{e}=10\;\mu m$.

Figure 9 and Figure 10 show areas of particles above the cut-off level determined automatically and on the basis of the average binder level (the methodology for calculating the cut-off levels for particles and pores based on the average level of the binder is presented later in the article).

For two grinding wheels, their topography was analyzed before starting the grinding process and after removing a certain volume of material. In order to facilitate the comparison of the results obtained with the two analyzed methods of determining the cut-off level, three groups of grains were distinguished (circled) for each of the grinding wheels. In both presented cases, the cut-off level determined automatically on the new grinding wheel (the specific material loss $V^{\prime }=0\;{\mathrm{mm}}^{3}/\mathrm{mm}$) was clearly “higher” than on the used grinding wheel. The determination of the cut-off level in relation to the average bond level resulted in the fact that the cut-off level at different stages of the grinding wheel wear varied to a lesser extent. As a result, it was possible to more reliably determine changes in height and volume parameters of particles due to wear.

## 6. Segmentation of Grains, Areas of Sticking, and Pores on GWAS Topographies

Segmentation of grains into the GWAS topography consisted of separating the measured point cloud particles located above the appropriately selected cut-off level. The particles on the measured object corresponded to the abrasive grains or their highest fragments and the areas of sticking, which were associated with gumming up of grinding wheels. In turn, the segmentation of the pores on the measured topographies was associated with the emergence of areas from these topographies that constitute cavities in the binder due to their unevenness or resulting from grain breaking.

**Figure 11:**
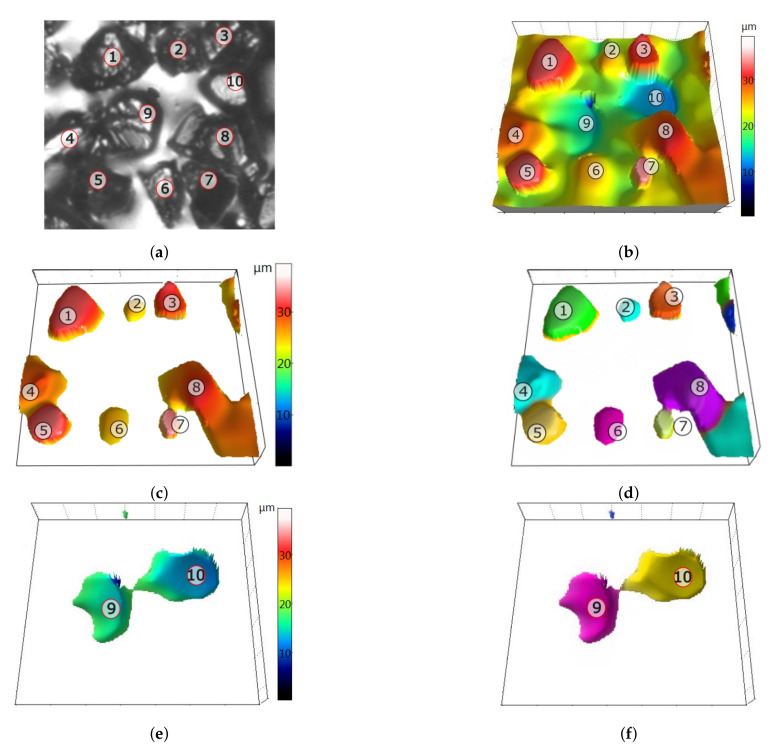
An example of particle and pore segmentation from the measured topography: (**a**) an image of a replica fragment with dimensions of 140μm × 140μm, (**b**) the corresponding map of the topography, (**c**) areas of particles above the cut-off level, (**d**) separated particles, (**e**) areas of pores below the cut-off level, and (**f**) isolated pores.

The most detailed information about the GWAS can be obtained when one particle corresponds to only one grain or stagnation area. Likewise, the pores observed should represent single cavities. To follow the aim of segmentation, the cut-off levels for particles and pores were not carried out at the designated average level of the binder. In the case of particles, the value of the mean level of the binder increased by 10μm was taken as the value that cuts off some of the points for further analysis. The “elevation” of the cut-off level compared to the average level of the binder served to remove more measuring points corresponding to the binder and low-lying portions of the grains, which improved the quality of grain recognition (Figure 11).

The purpose of isolating deep depressions on the measured topographies and determining their parameters was to provide information on the processes of grain extraction and the collection of chips and material derived from grains on the GWAS. The cut-off level below which the recesses were analyzed was set at the mean level of the binder reduced by 5μm. The “lowering” of the cut-off level compared to the average level of the adhesive served to improve pore separation by removing relatively shallow depressions resulting from uneven surfaces of the binder.

**Figure 12:**
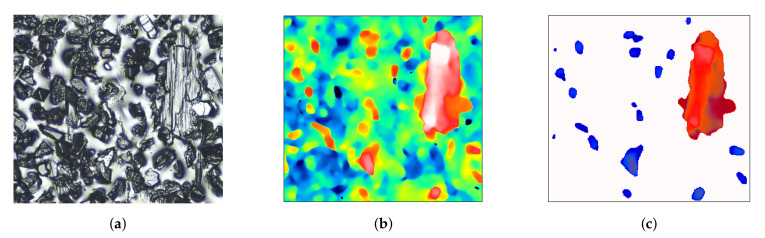
Example of grain-type and stick-type particles: replica image (**a**), topography map (**b**), grain-type particles (blue), and stick-type particles (red) (**c**).

After the cut-off levels for particles and pores had been established, the *Threshold* command was used to detect these elements. During the analysis of particles, they were divided into two categories (Figure 12):

- Particles of type “particles”: particles with an area in the range $\left[100,2500\right]\;\mu m^{2}$,
- Particles of type “sticking”: particles with an area bigger than $2500\;\mu m^{2}$.

The limit values of the particles’ surface area for the above-mentioned categories were determined on the basis of the topography analysis of new grinding wheels. Limiting the size of the “grains” above 100μm was to eliminate any residual binder, peaks resulting from the optical measurement system used, and peaks of low grains with low cutting potential. The value distinguishing “grains” from “sticking” was due to the fact that on each of the examined fragments of the topography of new grinding wheels, at least 99% of grains had a surface area smaller than 2500μm.

## 7. The Results of the Grinding Wheel Topography Tests During the Service Life

In the previous sections, the methodology of segmentation of the areas of abrasive grains, sticking, and pores was presented, including the determination of the average level of the binder using the proposed method. The results of the research on the active surface topography of the grinding wheel throughout its life are presented for a grinding wheel operating with the following adjustable parameters: $v_{s}=20\;m/s$ (for the maximum wheel diameter, $d_{s}=100\;\mathrm{mm}$), tangential feed speed $v_{w}=4250\;\mathrm{mm}/\min$, and grinding depth $a_{e}=10\;\mu m$. The moment when an intensive increase in the grinding force was observed was assumed as the end of the grinding wheel’s life. The tested grinding wheel was completely worn after achieving a specific material loss equal to $V^{\prime }=1751\;{\mathrm{mm}}^{3}/\mathrm{mm}$. The GWAS topography tests were carried out five times—at different stages of wear. Each time, six measurement areas were measured on the GWAS replicas.

**Figure 13:**
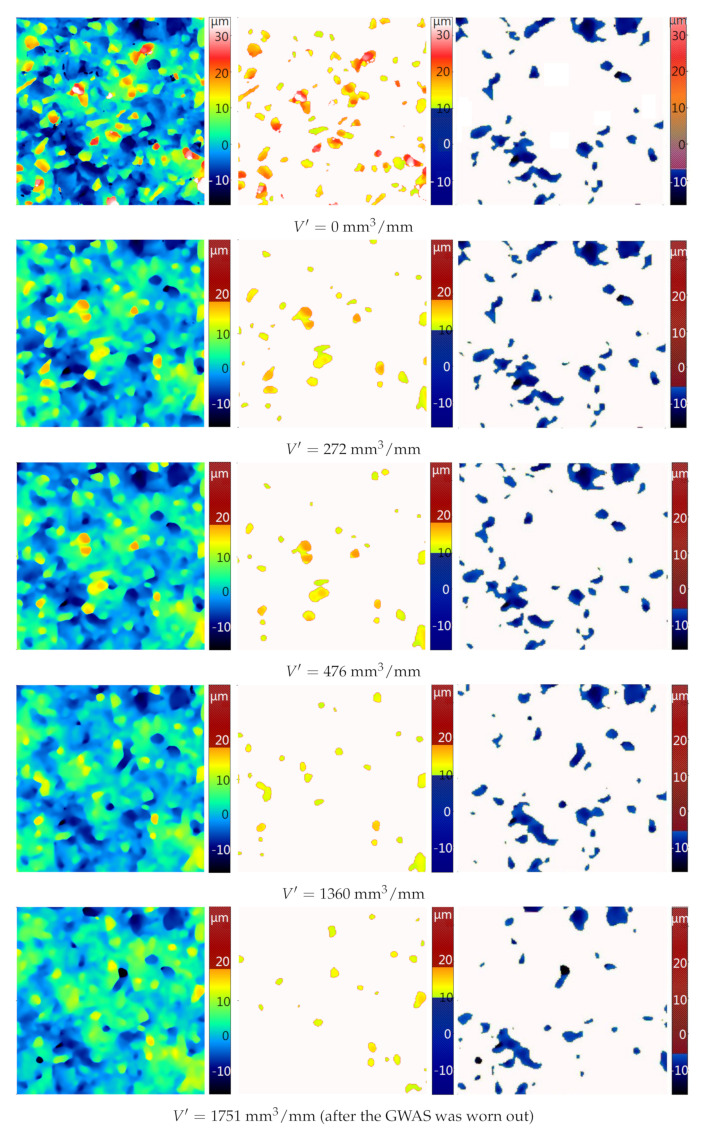
GWAS topography maps (**left**), corresponding particles maps above the cut-off level (**center**), and pore maps (**right**) for different values of the actual material loss $V^{\prime }$.

Figure 13 shows the fragments of the measured surface (0.5 mm × 0.5 mm) after removing various volumes of material. The height of the grains above the binder and the number of grains above the cut-off are visible as the volume of material removed increases. The changes in the depression areas in the binder are more difficult to notice. This is due to the fact that these changes occur mainly due to the removal of grains from the binder, which took place relatively rarely. The analysis of topography maps and microscopic images of GWAS replicas showed that the dominant type of wear was grain breaking. Hence, the differences in images and parameters of particles are more visible.

**Figure 14:**
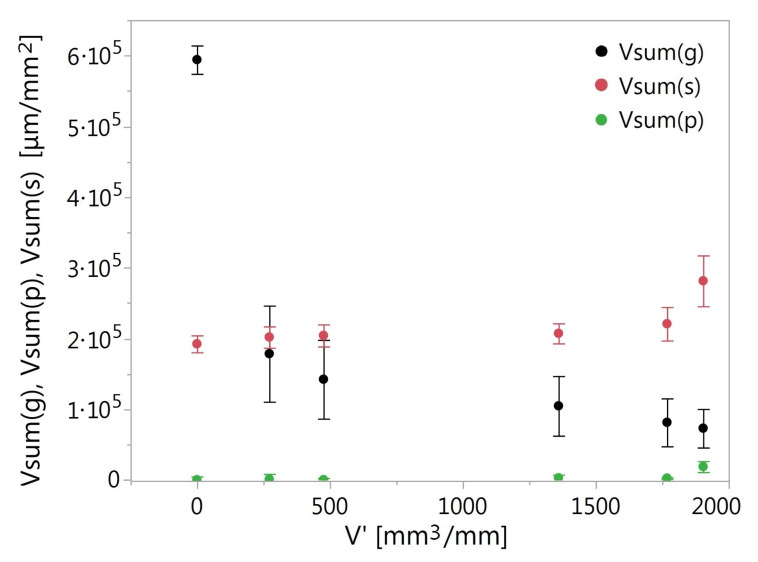
Grain volume (*Vsum*(*g*)), pore volume (*Vsum*(*p*)), and sticking area volume (*Vsum*(*s*)) per unit surface after removing different volumes of material.

The segmentation of particles and pores allowed for the determination of quantitative parameters of these elements. An example of a quantitative analysis of the GWAS at various stages of consumption is presented on the basis of volumetric parameters, i.e., grain volume (*Vsum(g)*), pore volume (*Vsum(p)*), and sticking area volume (*Vsum(s)*) per unit surface (Figure 14).

At the beginning of the grinding wheel’s operation, the grain volume decreased very intensively. The wear of the grains occurred mainly due to their chipping, as the pore volume increased slightly during this time. The largest pore volume gradient was observed at the end of the grinding wheel’s life. Therefore, it can be concluded that, at that stage of grinding wheel’s operation, the extraction of the grains took place with the greatest intensity. During the life of the wheel, $552\cdot 10^{3}\;\mu m^{3}/{\mathrm{mm}}^{2}$ of the abrasive was lost. The pore volume increased by about $79\cdot 10^{3}\;\mu m^{3}/{\mathrm{mm}}^{2}$. After the wheel was completely worn out, there was $35.5\cdot 10^{3}\;\mu m^{3}/{\mathrm{mm}}^{2}$ of the material that acted as a sealing material in the process of gumming up of the investigated GWAS.

More examples concerning the application of the proposed methodology of determining the cut-off level and segmentation of grains, areas of sticking, and pores on a GWAS in the context of grinding wheel performance are presented in [69]. That paper describes the results of research related to determination of GWAS parameters that are particularly sensitive to wear, including the parameters of grains, areas of sticking, and pores segmented using the developed methodology. It also presents the established mathematical relationships between the grinding process parameters and the specific material loss as well as a few selected GWAS parameters.

## 8. Conclusions

Based on the research on the active surface topography of a single-layer grinding wheel with a binder applied with the galvanic method and with cBN coating, the following conclusions can be drawn:

- Based on the measurement of the GWAS topography, it is possible to observe changes in the microgeometry of the grinding wheel occurring as a result of wear, and to perform a qualitative and quantitative analysis based on horizontal, height, and volume characteristics.
- The developed methodology for GWAS measurement allowed for the measurement of a GWAS in approximately the same places on the grinding wheel at different stages of wear. For the four surveyed areas, the common area accounted for 94% of their area.
- The analysis of particles above the specified cut-off level and pores below the cut-off level provides quantitative information, including height and volume information, on such characteristic GWAS elements as: abrasive grains, cavities in the binder caused by grain breakout, and areas of sticking.
- When analyzing changes in the GWAS topography areas related to grains, deep cavities, and sticking areas associated with gumming up of GWAS, which occur as a result of wear, it is important that the same fragments of these elements are constantly separated at different stages of wear. The cut-off levels for particles and pores should be at the same position on the wheel.
- The developed algorithm for determining the average level of the binder, against which the cut-off levels for particles and pores were determined, allows one to obtain more useful information about particles and pores analyzed in terms of grinding wheel wear than the available algorithm of automatic determination of the cut-off level in commercial software.
- Among the particles, there are areas corresponding to abrasive grains and sticking areas corresponding to gumming up of the GWAS. The categorization of particles into “grains” and “sticking” was established based on the surface area of the particle. The limit value for grain differentiation and sticking in the case of a grinding wheel with grain number B35 was set at 2500μm.
- For a grinding wheel with grinding speed $v_{s}=20\;m/s$ (for a maximum grinding wheel diameter of $d_{s}=100\;\mathrm{mm}$), feed rate of $v_{w}=4250\;\mathrm{mm}/\min$, and grinding depth of $a_{e}=10\;\mu m$, the largest volume of grains were broken in the initial period of the grinding wheel’s operation. The extraction of grains from the bond was most intense at the end of the grinding wheel’s life.

## Figures and Tables

**Table 1:** Parameters for measuring topography on the Alicona Focus Variation microscope.

Lens	×20
Single imaging field	0.71 mm × 0.54 mm
Number of imaging fields in the X and Y axes	4 × 5
Measurement area	2.35 mm × 2.59 mm
Analysis area	2.25 mm × 2.50 mm
Horizontal resolution	5 μm
Vertical resolution	100 nm
Sampling step	0.44 μm × 0.44 μm

**Table 2:** Mean value, standard deviation, and quartiles $Q_{1}$, $Q_{2}$, and $Q_{3}$ computed for the analyzed GWAS areas.

	OA Error [%]	AT Error [%]	Improvement [%]
mean	9.35	57.64	48.29
std	5.6	46.17	42.87
min	0.67	14.76	7.61
*Q*_1_	5.34	25.85	14.94
*Q*_2_	9.17	43.92	34.75
*Q*_3_	13.95	80.36	71.24
max	17.65	141.00	125.74

## Abbreviations

The following abbreviations are used in this manuscript:

AT	Automatically determined
BAC	Bearing area curve
cBN	Cubic boron nitride
GWAS	Grinding wheel active surface
ISO	International Standards Organization
OA	Developed algorithm
ST	Surface texture
SLGW	Single-layer grinding wheel
